# Diagnosed by Reversibility: Unmasking Propofol-Related Infusion Syndrome in a Critically-Ill Elderly Male

**DOI:** 10.7759/cureus.23504

**Published:** 2022-03-26

**Authors:** Tewodros Ayele, Ebubechukwu Ezeh, Leesah Al-Qawasmi, Onyinye S Ugonabo, John Saylor, Larry Dial

**Affiliations:** 1 Internal Medicine, Marshall University Joan C. Edwards School of Medicine, Huntington, USA

**Keywords:** anesthesia, intensive care, metabolic acidosis, critically ill elderly patients, propofol, pris, propofol infusion syndrome

## Abstract

Propofol-related infusion syndrome (PRIS) is an uncommon complication resulting from prolonged propofol use. Common clinical presentations include metabolic acidosis, cardiac arrhythmias, and renal complications. The mortality rate is high if it is not recognized in time. There is no antidote to propofol. Initial treatment involves discontinuing ongoing propofol use and providing supportive measures. The reversal of clinical and laboratory features upon discontinuation of propofol provides a basis for retrospective diagnosis or PRIS. In severe cases, ultrafiltration may be utilized.

## Introduction

Propofol is a widely used sedative-hypnotic agent for induction of general anesthesia and sedation in the ICU. However, it is associated with a rare but highly fatal condition known as propofol-related infusion syndrome (PRIS). Common manifestations of the syndrome include metabolic acidosis, arrhythmias, acute renal failure, rhabdomyolysis, hyperkalemia, cardiovascular collapse, and hypertriglyceridemia [1]. The incidence of PRIS is unknown but is estimated to be less than 1% [2].

Central to the pathogenesis is an imbalance between energy demand and supply, leading to cardiac and skeletal muscle necrosis. In addition, propofol impairs free fatty acid utilization and mitochondrial activity resulting in the accumulation of free fatty acids (FFA) with proarrhythmogenic properties [3].

Occurrence of the syndrome, as well as its severity, appears to be dose-dependent, with most cases occurring in patients who received a propofol dose in excess of 5 milligrams per kilogram per minute (mg/kg/hr) or 80 micrograms per kilogram per minute (μg/kg/min) for at least 48 hours. 

Here we report a 73-year-old gentleman who was admitted for management of very severe COVID-19 pneumonia with a hospital course complicated by PRIS. 

## Case presentation

A 73-year-old unvaccinated male was transferred to our facility for further management of complications of COVID-19 pneumonia. He had a past medical history of chronic obstructive pulmonary disease and type 2 diabetes mellitus. He had presented to an outside facility four days prior with shortness of breath, fever, and a productive cough. Initial arterial blood gas (ABG) showed pH 7.40, partial pressure of carbon dioxide (PCO2) 35 millimeters of mercury (mmHg), and partial pressure of oxygen (PO2) of 82 mmHg. Serum creatinine was 0.9 milligram per deciliter (reference range 0.8 to 1.2 mg/dl), and lactic acid was 1.1 millimole per liter (reference range 0.5 to 2.2 mmol/L). On the second day, he was intubated on account of worsening hypoxemic respiratory failure. Propofol (at 5.2 mg/kg/hour) and fentanyl were started for sedation and analgesia, respectively. Remdesivir, dexamethasone, and antibiotics were also started. He was then referred to our facility. 

On presentation to our facility, arterial blood gas (ABG) showed pH 7.20, PCO2 47 mmHg, and PO2 of 64 mmHg. Labs showed lactic acid 4 mmol/L, triglyceride 539 milligram per deciliter (reference range < 150 mg/dl), phosphorus 6.2 mg/dl (reference range 2.3 to 4.7), and creatinine 2.5 mg/dl. Two days later, serum creatinine had increased to 3.3 mg/dl, pH declined further to 7.01, and triglyceride had increased to 1096 mg/dl. Creatine phosphokinase (CPK) was slightly elevated at 421. Remdesivir was discontinued because the estimated glomerular filtration rate (eGFR) was now 20 milliliters per minute (mL/min/1.73m2). A transthoracic echocardiogram showed normal ejection fraction and chamber sizes and functions.

Despite starting an insulin drip, triglycerides level continued to increase, peaking at 1430 mg/dl. At this time, propofol-related infusion syndrome was suspected, and propofol was discontinued. This was after he had been on it for about 72 hours. Midazolam was started in place of propofol. Within the next two days, triglycerides had reduced to 130 mg/dl, with pH improving to 7.36. Creatinine, lactic acid, phosphorus, and eGFR gradually normalized. The patient went on to have a prolonged hospital course due to complications from his COVID-19 infection.

## Discussion

Propofol-related infusion syndrome is characterized by protean clinical and laboratory manifestations. There is also significant variability in the presentation features of the syndrome. According to a systematic review of 168 cases of PRIS reported in the literature, the most common clinical manifestation is metabolic acidosis which is reported in 80% of cases, followed by ECG changes [4]. Our patient had major features of PRIS, including metabolic acidosis, acute renal failure, and hyperlipemia. He was on propofol for about 72 hours which is beyond the 48 hours period of exposure required to develop symptoms. 

Propofol-related infusion syndrome is a multifactorial syndrome; the priming factor is a critical illness, and propofol, catecholamines, and steroids contribute as triggering factors [3]. Cumulative dose of propofol is associated with the number of clinical features and organs systems affected by the syndrome [4]. As the clinical features are protean and a high degree of overlap with manifestations of underlying critical illness, a high index of suspicion is crucial in the prevention, early identification, and treatment of this condition. 

There is no antidote for propofol, and management depends on the clearance of propofol from the body. Propofol is metabolized in the liver, and its water-soluble metabolites are excreted through the kidneys. Although it cannot eliminate the highly lipophilic parent drug, continuous hemofiltration can eliminate the toxic water-soluble propofol metabolites [5]. For those with severe forms of cardiovascular collapse, mechanical circulatory support, using ventricular assist devices (VADs) or extracorporeal membrane oxygenation (ECMO) can be deployed to temporarily support end-organ blood flow and oxygen delivery [6].

## Conclusions

Propofol is a commonly utilized sedative agent in critically ill adults. However, it is associated with a rare but highly fatal and potentially reversible complication called propofol-related infusion syndrome. Having a high index of suspicion with knowledge of clinical features and prompt discontinuation with switching to alternative agents such as midazolam could be effective in reversing the condition. 
